# Silencing of matrix metalloprotease-12 delays the progression of castration-resistant prostate cancer by regulating autophagy and lipolysis

**DOI:** 10.1590/1414-431X2024e13351

**Published:** 2024-03-18

**Authors:** Xiaoyu Zheng, Xiaoqin Xie, Wei Wang, Liang Wang, Bing Tan

**Affiliations:** 1 School of Clinical Medicine, Chongqing Medical and Pharmaceutical College, Chongqing, China; 2 Department of Clinical Laboratory, Chongqing Blood Center, Chongqing, China; 3 Department of Orthopedics, The People's Hospital of Yubei District of Chongqing City, Chongqing, China; 4 Department of Dermatology, Daping Hospital, Army Medical University, Chongqing, China; 5 Department of Urology and Medical Sciences Research Center, University-Town Hospital of Chongqing Medical University, Chongqing, China

**Keywords:** CRPC, MMP-12 Autophagy, Lipolysis

## Abstract

The complex pathogenesis of castration-resistant prostate cancer (CRPC) makes it challenging to identify effective treatment methods. Matrix metalloproteinase (MMP)-12 can degrade elastin as well as various extracellular matrix (ECM) components, which is associated with cancer progression. However, the relationship between MMP-12 and CRPC progression is poorly understood. In this study, we observed the effect of MMP-12 on the progression of CRPC and further explored its potential mechanism of action. High levels of MMP-12 were observed in patients with CRPC. We therefore developed cell co-culture and mouse models to study the function of MMP-12. Silencing MMP-12 in CRPC cells disrupted lipid utilization and autophagy marker expression via the CD36/CPT1 and P62/LC3 pathways, respectively, leading to reduced CRPC cell migration and invasion. Moreover, animal experiments confirmed that MMP-12-knockdown CRPC xenograft tumors exhibited reduced tumor growth, and the mechanisms involved the promotion of cancer cell autophagy and the inhibition of lipid catabolism. According to our results, MMP-12 played important roles in the progression of CRPC by disrupting adipocyte maturation and regulating cancer migration and invasion via the modulation of autophagy and lipid catabolism pathways.

## Introduction

Prostate cancer is one of the leading causes of cancer-related death among men. Approximately 54% of patients with prostate cancer in China have distant metastases, including bone and abdominal organ metastases (1). At the time of diagnosis, the five-year relative survival rate of patients without metastasis has already decreased from 80 to 30% (1,2). Androgen deprivation therapy (ADT) is the primary treatment for patients diagnosed with prostate cancer, but almost all patients develop drug resistance after two years of treatment and progress to castration-resistant prostate cancer (CRPC) (3). Once a patient enters the CRPC stage, the prognosis is poor. Although the use of endocrine therapeutic agents partially inhibits the progression of CRPC, the development of drug resistance is inevitable (4,5). This phenomenon may be related to the mechanism underlying CRPC progression.

Moreover, periprostatic adipose tissue (PPAT) is closely associated with prostate cancer progression because it surrounds the prostate (6). Mature adipose tissues secrete hormones, cytokines, chemokines, and inflammatory factors that are critical components of the tumor microenvironment (7). During prostate cancer progression, adipocytes from PPAT promote the outward spread of prostate cancer cells, and this is an essential step for cancer dissemination (8). Measurements of periprostatic adiposity could help predict the time of CRPC onset (9). However, the mechanism by which PPAT influences CRPC occurrence and progression has not been determined.

Matrix metalloproteinases (MMPs) are a class of protein hydrolases that degrade extracellular matrix (ECM) components and are involved in tumorigenesis, development, invasion, and metastasis (10). There are more than 23 members of the MMP family in humans, and these members are identified according to their different structural domains. Among the MMP family members, MMP-12 was first described in mouse peritoneal macrophage cultures; it is produced by macrophages and degrades elastin and various ECM components (11). The MMP-12 protein participates in inflammatory processes and is a vital regulator of tumor growth and occurrence (12,13). MMP-12 expression by prostate cancer cells is associated with bone marrow stromal cell-induced invasion (14), but understanding its role in depth requires further study. In addition, autophagy is an important pathway of protein degradation in eukaryotic cells (15). Autophagy provides nutrients and energy for cell survival by degrading unnecessary or dysfunctional intracellular components (16). As a consequence of the cellular environment, autophagy can either be deleterious or protect against prostate cancer (17). It has been found that co-culture of prostate cancer cells with adipocytes reduced autophagic activity in PC3 cells, suggesting that autophagy in cancer and adipocytes may be closely related (18). Therefore, it is necessary to investigate whether MMP-12 promotes CRPC progression through a mechanism related to cell autophagy and lipid catabolism.

In our preliminary clinical observation, we found that MMP-12 was highly expressed in CRPC but was hardly expressed in patients with benign prostatic hyperplasia (BPH) and that high expression of MMP-12 was associated with poor pathological stage. These findings confirmed that MMP-12 plays important roles in the progression of CRPC. Furthermore, via *in vivo* and *in vitro* experiments, we investigated the function of MMP-12 in CRPC lipid catabolism and autophagy. These data suggested that MMP-12 deficiency can attenuate the migration and invasion of CRPC cells by regulating lipid catabolism and autophagy.

## Material and Methods

### Clinical samples

The clinical tissues and blood samples from BPH, androgen-dependent prostate cancer (ADPC), and CRPC were collected from patients at the University-Town Hospital of Chongqing Medical University. The pathological examination confirmed the tissue type. All procedures were performed with the patients’ informed consent and approved by the Ethics Committee of the University-Town Hospital of Chongqing Medical University. The study strictly complied with the 1964 Helsinki Declaration and its later amendments or comparable ethical standards.

### Immunohistochemistry

Clinical tissues and mouse tumors were fixed with 4% paraformaldehyde, dehydrated with different ethanol concentrations, embedded in paraffin, and then sliced (4 μm). Sections were dewaxed with xylene, rehydrated with ethanol of a gradient concentration, and rinsed with distilled water. Antigen repair was then performed with 0.01 M sodium citrate buffer (pH 6.0) for 30 min, treatment with 3% H_2_O_2_ for 5 min, and permeabilization with 0.1% TritionX-100 in PBS for 15 min. The primary antibodies, including anti-human MMP-12 antibody (1:300; MAB919; R&D Systems, USA) and anti-mouse LC3B antibody (1:200; 83506; Cell Signaling Technology, USA) were incubated overnight, and the tissue sections were incubated with secondary antibodies containing horseradish peroxidase for 45 min. The sections were stained with hematoxylin-eosin (HE) and made transparent with xylene after dehydration. According to staining intensity, sections were scored as 0=no staining, 1=mild staining, 2=moderate staining, and 3=intense staining. Immune response rate was recorded as 0 (0% immune response cells), 1 (<5%), 2 (5-50%), 3 (>50%). The final score was obtained by combining staining intensity with immune response rate. A score equal to or greater than 3 was considered positive staining.

### HE staining

The clinical tissue sections were fixed in a 4% paraformaldehyde solution. Then, they were dehydrated with ethanol, made transparent with xylene, embedded in paraffin, and sliced (4 μm). Later, they were stained with HE, dehydrated and made transparent again, and sealed with neutral gum. The histopathological changes were observed with an optical microscope (Nikon E100, Japan).

### ELISA assay

The defrosted human serum and cell medium were tested by ELISA kit according to the instructions (ab255715, Abcam, UK), and an enzyme-labeled instrument (Tecan Infinite 200, Switzerland) was used to detect the protein content of MMP-12.

### Cell culture and establishment of co-culture models

C42B and PC3 cell lines were acquired from the American Type Culture Collection and kindly donated by the Key Laboratory of Clinical Laboratory Diagnostics, Chongqing Medical University. Specifications of PC3 are category number: CRL-1435; species: human; source: prostate adenocarcinoma grade IV; morphology: epithelial; characteristics: androgen-independent. Specifications of C42B are category number: CRL-3315; species: human; source: prostate cancer bone metastatic; morphology: epithelial-like; characteristics: androgen-independent. Cell lines were cultured in Dulbecco's modified Eagle's medium/F-12 medium (Gibco, USA) containing 10% fetal bovine serum (FBS, Gibco), 2 mM glutamine, and 100 U/mL streptomycin/penicillin (Life Technologies, USA).

3T3-L1 preadipocytes (FuHeng Biotech, China) were cultured in DMEM high-glucose medium (10% FBS, Gibco). Cell differentiation was induced by DMEM high-glucose medium (10% FBS, 1% penicystreptomycin, 10 μg/mL insulin, 0.5 μmol/L 3-isobutyl 1-methyl xanthine, 1 μmol/L rosiglitazone, 1 μmol/L dexamethasone), and after 48 h, it was treated with an insulin-induced medium (10 μg/mL INS). The complete medium was used without an inducer after 72 h, and the liquid was changed every 2 days until the massive accumulation of fat droplets.

MMP-12 siRNA (siMMP-12) and non-targeted siRNA (siNT) were synthesized by Shanghai Gima Pharmaceutical Technology (China). CRPC cells were maintained for 48 h after being transfected with 50 nM of siRNA by using Lipofectamine^TM^ 2000 reagent (Invitrogen, USA) and used for further investigations.

### Cell migration and invasion assays

Transwell assay was used to detect the migration and invasion ability of CRPC cells. In the migration assay, cells were inoculated in the upper chamber without Matrigel coating. In the invasion experiment, the upper chamber was pre-coated with 5% Matrigel matrix glue (Corning BD 356234, China), 4×10^4^ CRPC cells were added to the upper chamber lined by a membrane with 8-μm pores, and then 500 μL serum-free medium was added. Adipocytes were maintained in the lower chamber along with the FBS-enriched medium. After incubation for 48 h, the invaded cells were fixed with 4% paraformaldehyde for 15 min and then stained with crystal violet. Five visual fields were randomly selected under an inverted microscope (Zeiss, Germany) to count the number of invaded cells.

### Oil red O staining

Adipocytes were stained by Oil Red O Set Box (BP037, Biossci, China). The mature adipocytes were dyed with a working solution for 8 min. The excess solution was slightly washed with 60% isopropyl alcohol and then with distilled water.

### Western blot assay

Protein was extracted in RIPA buffer, and the protein concentration was detected by the BCA kit (Beyotime P0011, China). After gel electrophoresis, PVDF membranes were incubated with primary and secondary antibodies, and the primary antibodies included anti-aP2 (1:500; 2H3-1G10; Novusbio, USA), anti-MMP-7 (1:500; abs146189; Absin, China), anti-MMP-9 (1:1000; ab76003; Absin), anti-MMP-12 (1:1000, ab52897; Absin), anti-CPT1 (1:1000; #97361; Cell Signaling Technology), anti-CD36 (1:1000; ab252922; Abcam), anti-P62 (1:1000; ab109012; Abcam), anti-LC3A/B (1:1500; #12741; Cell Signaling Technology), and GAPDH (1:5000; YM3029; Immunoway, USA). Images were processed by ECL chemiluminescence.

### Reverse transcription and real-time PCR

Total RNA was extracted from cells by using TRIzol reagent. The samples were reverse-transcribed into cDNA using the PrimeScriptTM RT kit (Takara, Japan). Real-time PCR was performed according to the instructions of the SYBR Green PCR Master Mix (Invitrogen). Primers for MMP12 (forward-GAACAGCTCTACAAGCCTGGAA, reverse-TCTCCAGGTAGATGTGTCCAGT), aP2 (forward-AAGAAGTGGGAGTGGGCTTTG, reverse-CTCTTCACCTTCCTGTCGTCTG), β-actin (forward-TACCTCATGAAGATCCTCACC, reverse-TTTCGTGGATGCCACAGGAC).

### Immunofluorescence

C42B cells (5×10^4^) were inoculated onto culture plates, fixed with 4% paraformaldehyde, cleaned with PBS, infiltrated with 0.1% Triton X-100 for 5 min, and then blocked with 1% BSA for 1 h. Primary antibody LC3B (1:100; ab192890; Abcam) was incubated at 4°C overnight, then incubated with a secondary antibody (1:200; ab150081; Abcam) at room temperature for 1 h, and re-stained with DAPI for 5 min. An anti-fluorescence quencher (Beyotime P0128S, China) was added to the slide and photographed by an inverted fluorescence microscope (Zeiss, Germany).

### Animal xenograft assay

C42B and PC3 cells (1×10^7^) were implanted into the bilateral subcutaneous inguinal region of a male nude mouse. Mice (8 weeks old) were divided into the control group, the siNT group, and the siMMP-12 group (5 mice/group). The tumor volume was calculated every week by the following formula (4/3) × 3.14 × (length/2) × (width/2) × (height/2). After 3 weeks, the mice were anesthetized by an intraperitoneal injection of sodium pentobarbital solution (35 mg/kg), tumors were surgically removed. Then, the tumor and body weight were measured by electronic balance. The Ethics Committee of Chongqing Medical and Pharmaceutical College approved animal procedures. All animal experiments were carried out in accordance with the National Institutes of Health Guide for the Care and Use of Laboratory Animals (NIH Publications No. 8023, revised 1978).

### Statistical analysis

GraphPad Prism 9 software (GraphPad Software Inc., USA) was used for mapping and data analysis. Experiments were performed in triplicate at least three times. All data are reported as means±SD. Unpaired multiple *t*-tests were used to test for significance, and P<0.05 was considered statistically significant.

## Results

### MMP-12 was highly expressed in CRPC tissues

**Figure 1:**
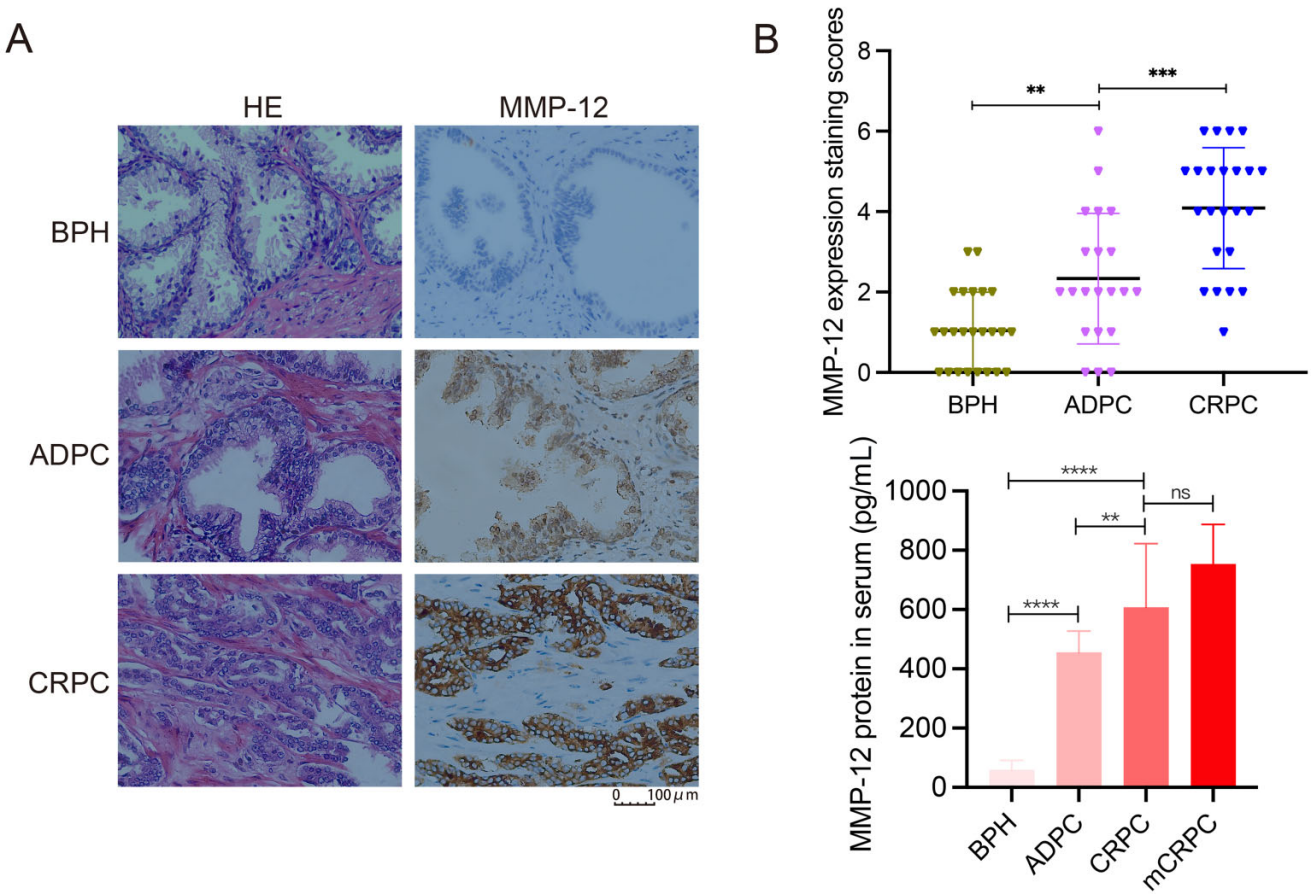
MMP-12 expression level becomes increasingly elevated as prostate cancer progresses: **A**, The MMP-12 protein expression (left) and quantification (right) in patients with pathological stages of benign prostatic hyperplasia (BPH), androgen-dependent prostate cancer (ADPC), and castration-resistant prostate cancer (CRPC) was determined by immunohistochemistry (scale bar 100 μm). **B**, The concentration of MMP-12 protein in the serum of the different groups of patients was determined by ELISA. Data are reported as means±SD. **P<0.01, ***P<0.001, and ****P<0.0001 unpaired *t*-test. ns: not significant.

To determine the expression profile of MMP-12 in clinicopathological tissues, we collected surgical specimens from BPH, ADPC, and CRPC patients. Histopathological examinations confirmed the increased aggressiveness of cancer morphology. The MMP-12 protein was strongly expressed in cancer cells from the CRPC group, light MMP-12 staining was observed in the ADPC group, but the BPH samples exhibited very little staining (Figure 1A). The levels of the MMP-12 protein in patient serum samples exhibited a similar pattern in these three groups. In many CRPC patients, the cancer had metastasized to other organs, such as the bone and lung, and this condition is called metastatic CRPC (mCRPC). In some cases, mCRPC patients had markedly higher MMP-12 levels than patients without metastases (Figure 1B).

Next, we assessed the correlation between MMP-12 expression and the clinicopathological characteristics of CRPC patients. As shown in Table 1, high MMP-12 levels were associated with an increased Gleason score (P<0.05). However, MMP-12 expression was not correlated with age, histological stage, organ metastasis, or blood prostate specific antigen (PSA) levels.

**Table 1:** Correlation between MMP-12 expression and the clinicopathological characteristics in castration-resistant prostate cancer (CRPC).

Variable	Specimens (n, %)	MMP-12		P value
		Positive	Negative	
Age (years)				
≤60	24 (66.67)	20	4	0.294
>60	12 (33.33)	9	3	
Histologic stage				
T1a-T2c	13 (36.11)	10	3	0.063
T3a-T4	23 (63.89)	16	7	
Gleason score				
≤7	15 (41.67)	9	6	0.041*
>7	21 (58.33)	17	4	
Organ metastasis				
Negative	20 (55.56)	10	10	0.839
Positive	16 (44.44)	7	9	
PSA level (ng/mL)				
≤10	9 (25)	8	1	0.657
>10	27 (75)	23	4	

PSA: Prostate specific antigen. *Statistically significant, Kappa test.

### Silencing MMP-12 in CRPC cells contributed to adipocyte maturation

**Figure 2:**
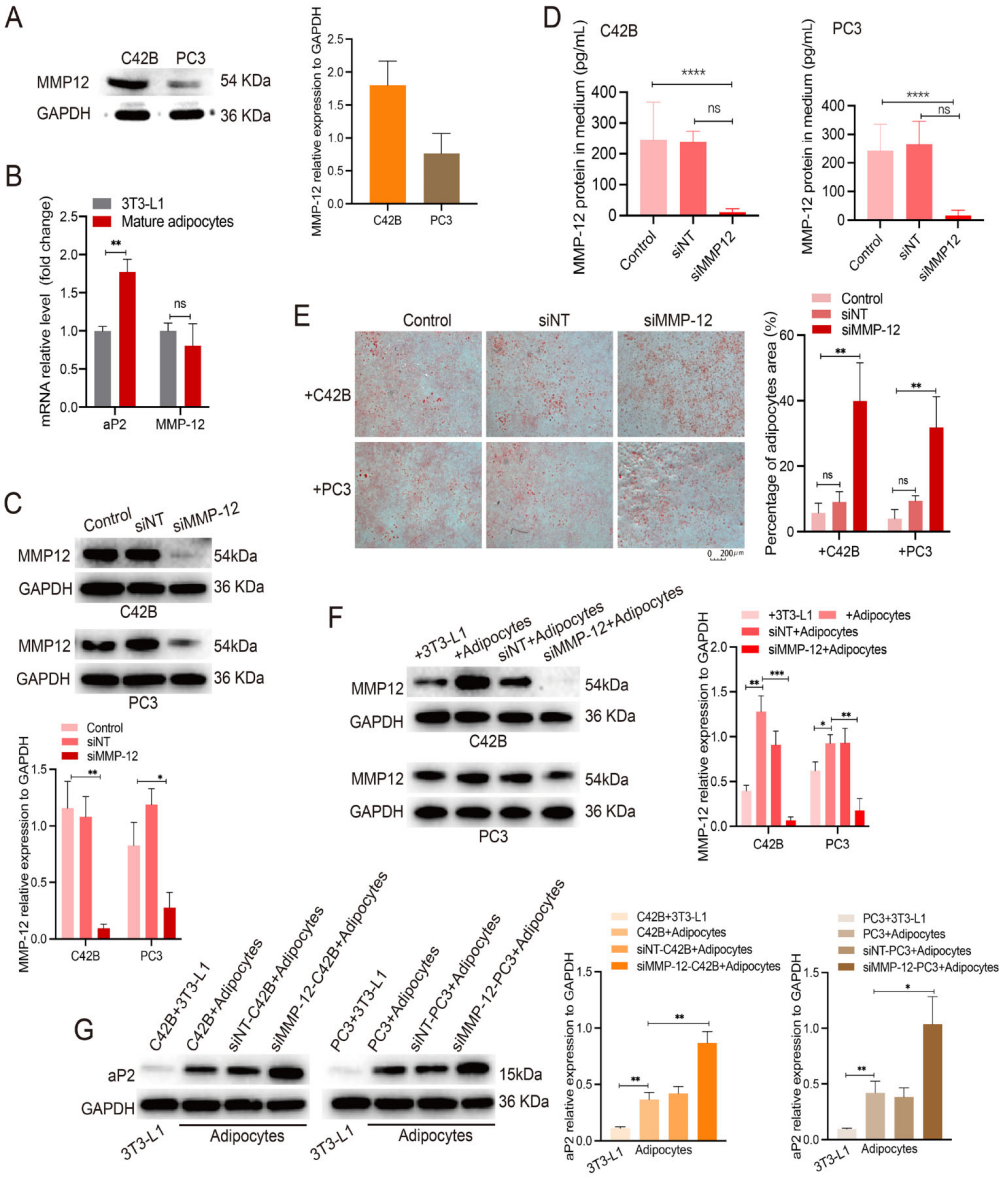
Silence of MMP-12 could maintain the adipocytes' mature state. **A**, The MMP-12 protein in C42B and PC3 cell lines was detected by western immunoblotting. **B**, Relative mRNA expression of aP2 and MMP-12 in preadipocytes 3T3-L1 and mature adipocytes. **C**, siRNA silenced MMP-12 (siMMP-12) protein expression in C42B and PC3 cell lines. **D**, The concentration of MMP-12 protein in the culture medium was measured by ELISA in both C42B and PC3 cell groups. **E**, The mature adipocyte area was validated by Oil red O staining in the co-culture system in both C42B and PC3 cell groups (scale bar 200 μm). **F**, MMP-12 expression was evaluated in C42B and PC3 cells cultivated with mature adipocytes compared with preadipocytes 3T3-L1, while it was silenced by siRNA treatment. **G**, aP2 expression was elevated in mature adipocytes compared with 3T3-L1 when co-cultivated with castration-resistant prostate cancer (CRPC) cells, and levels were even higher with siMMP-12 treatment. Results represent three independent experiments. Data are reported as means±SD. *P<0.05, **P<0.01, ***P<0.001, and ****P<0.0001 unpaired *t*-test. ns: not significant; siNT: non-targeted siRNA.

We measured MMP-12 expression in CRPC cell lines and found high levels of MMP-12 expression in C42B and PC3 cells (Figure 2A). aP2 protein (adipocyte protein 2 gene product) expression is typically used to assess full adipocyte differentiation (19); we also observed that neither preadipocytes nor mature adipocytes expressed MMP-12 (Figure 2B). Transfection of siRNA targeting MMP-12 into C42B and PC3 cells significantly downregulated the expression of MMP-12 (Figure 2C). Moreover, we observed a significantly decreased level of MMP-12 in the culture medium, which suggested that MMP-12 could be secreted by CRPC cells into the extracellular space (Figure 2D). These results prompted us to further study the effect of CRPC cells on the process of adipocyte maturation.

Since adipocytes are among the most crucial components of the tumor matrix, they may facilitate tumor progression by secreting adipocytokines and free fatty acids. We next established an *in vitro* cell co-culture model. Oil Red O staining demonstrated that a large percentage of mature adipocytes were present in the co-culture system after MMP-12 was knocked down in CRPC cells (Figure 2E). Interestingly, compared to preadipocytes, mature adipocytes promoted MMP-12 expression in CRPC cells, but this effect was inhibited by MMP-12 silencing (Figure 2F). Conversely, co-culture with siMMP12-CRPC cells enhanced the expression of the maturation marker aP2 in adipocytes (Figure 2G). These findings indicated that MMP-12 derived from CRPC cells may play vital roles in adipocyte immaturity, thereby enhancing the stemness of the tumor ECM.

### MMP-12 knockdown reduced CRPC cell migration and invasion via the CD36/CPT1 and P62/LC3 pathways

**Figure 3:**
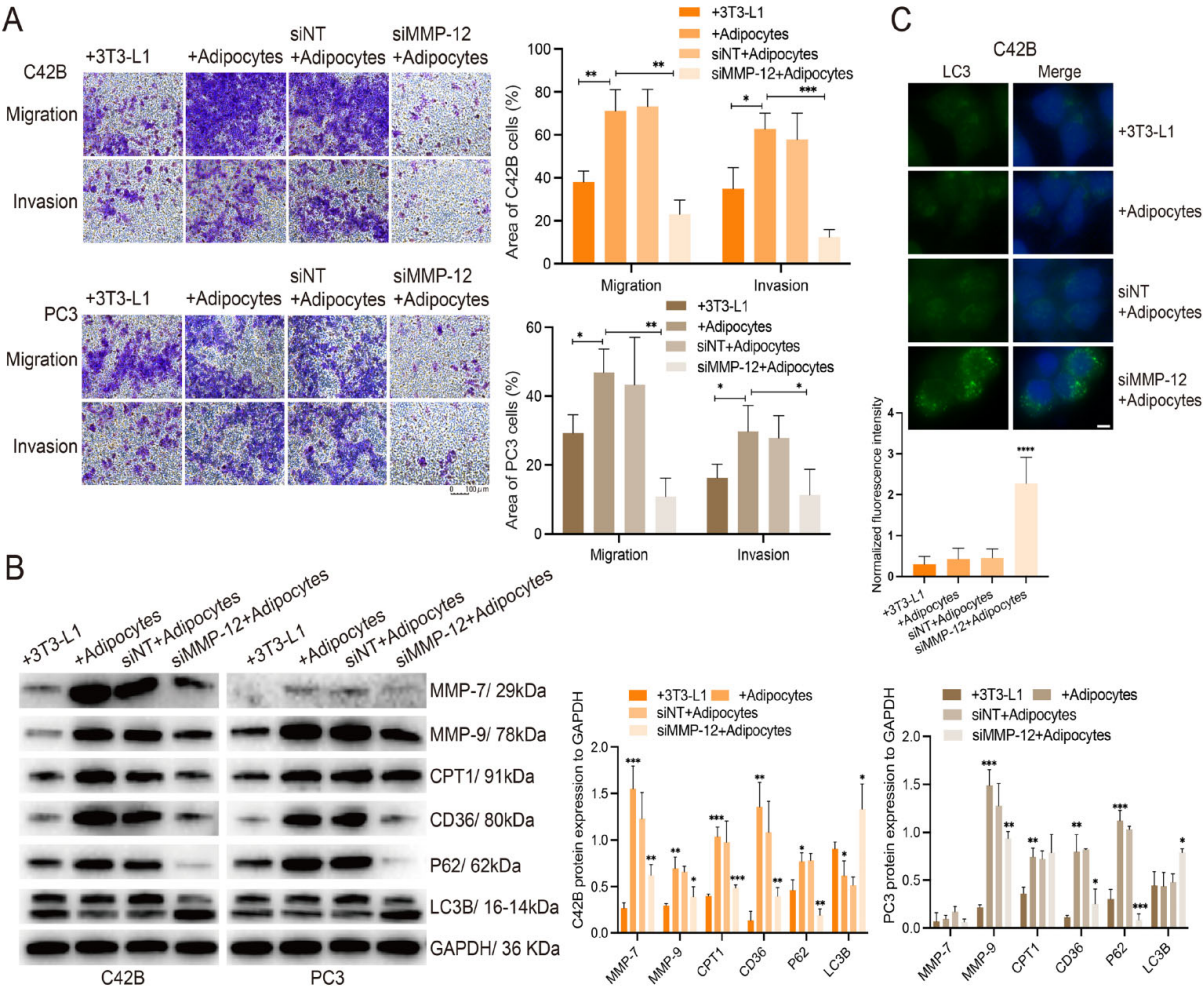
The silencing of MMP-12 attenuated castration-resistant prostate cancer (CRPC) cell migration and invasion by alleviating lipid catabolism and promoting autophagy. **A**, The migration and invasion of CRPC cells were evaluated by transwell assay without or with Matrigel-coated under a co-culture condition (scale bar 100 μm). **B**, Cancer invasion-related MMP-7 and MMP-9, lipolysis-related CD36 and CPT1, and autophagy-related P62 and LC3B were detected by western blotting in individual groups. **C**, The LC3 protein expression in co-cultured C42B cells was detected by immunofluorescence staining (scale bar 5 μm). Results represent three independent experiments. Data are reported as means±SD. *P<0.05, **P<0.01, ***P<0.001, and ****P<0.0001 unpaired *t*-test. siMMP-12: siRNA silenced MMP-12; siNT: non-targeted siRNA.

To determine changes in cancer cell migration and invasion in the absence of MMP-12, we performed transwell assays with or without Matrigel coating in the upper chamber. When adipocytes were seeded in the lower chamber, C42B and PC3 cells displayed greater migrative and invasive capacities than when preadipocytes were seeded in the lower chamber. Compared with the nontransfected group, the siMMP-12-transfected groups showed significantly inhibited migration and invasion of CRPC cells into the lower chamber (Figure 3A).

To understand the molecular mechanisms underlying the change in the malignant activity of CRPC cells, we co-cultured C42B or PC3 cells with 3T3-L1 cells or mature adipocytes for 72 h. MMP-7 and MMP-9 are known to be markers of prostate cancer invasion (20,21), and we observed elevated expression of these proteins (Figure 3B). CD36 is a protein in the cell membrane that is involved in lipid uptake, and CPT1 is an enzyme in the cytoplasm that is related to lipid oxidation. Co-culture with adipocytes improved cancer cell lipid catabolism through the CD36/CPT1 pathway. However, these effects were reversed after MMP-12 silencing (Figure 3B). Moreover, an immunofluorescence assay further confirmed that the expression of the autophagy-related protein LC3 was enhanced in siMMP-12-transfected C42B cells after they were cultured with adipocytes (Figure 3C). These results revealed that MMP12 affects CRPC cell lipid catabolism and autophagy via interaction with adipocytes.

### MMP-12-knockdown CRPC tumors exhibited reduced growth via the promotion of autophagy *in vivo*

**Figure 4:**
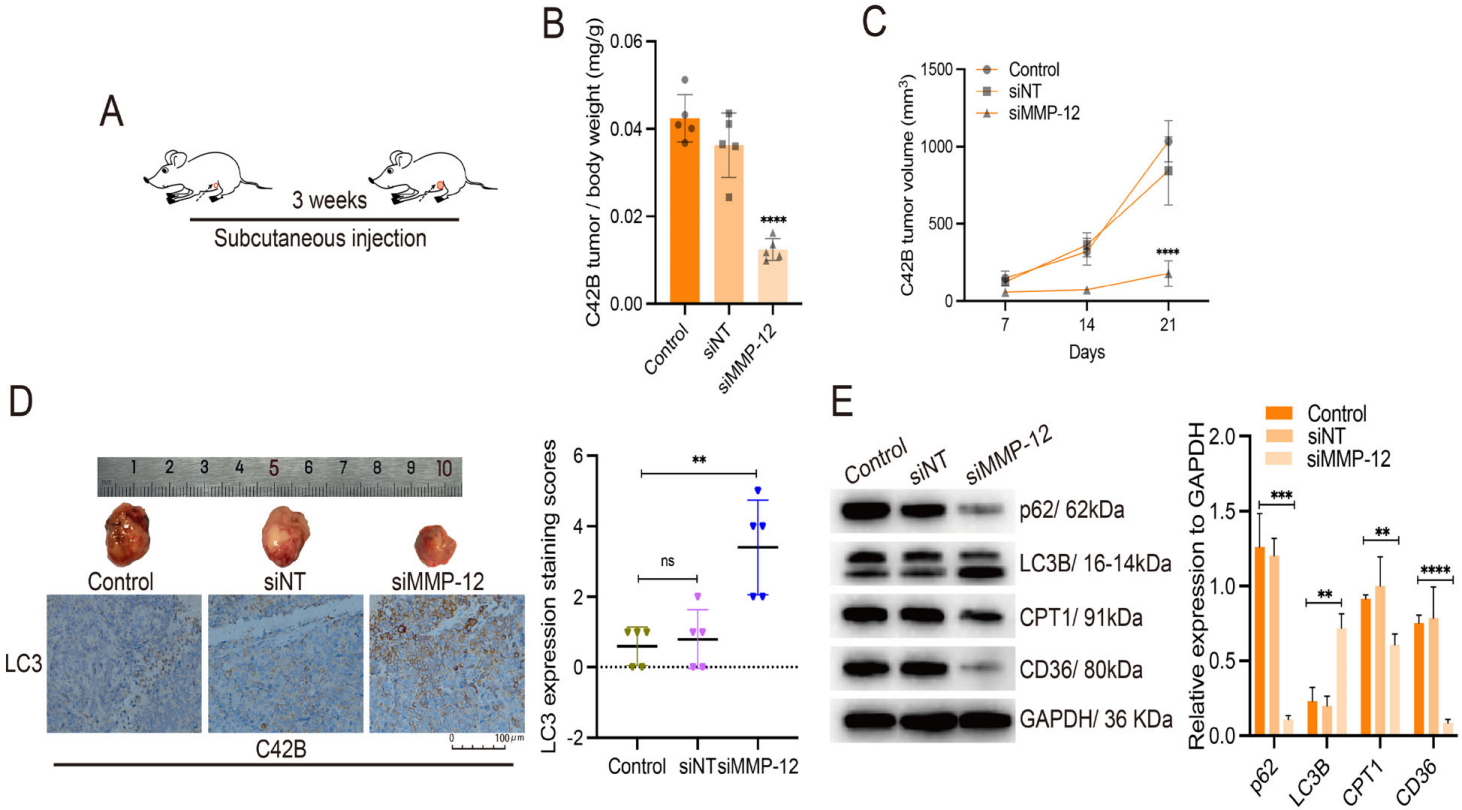
The loss of MMP-12 inhibited mice castration-resistant prostate cancer (CRPC) tumor growth. **A**, Timeline of the mouse experiment. **B**, Relative tumor weight was measured in different groups at the end of the experiment. **C**, Tumor volume was measured by caliper in these three groups at 7, 14, and 21 days. **D**, The C42B tumor gross appearances are shown and LC3 protein was detected and quantified by immunohistochemical staining (scale bar 100 μm). **E**, Autophagy-related P62 and LC3B and lipolysis-related CD36 and CPT1 were detected by western blotting in tumor tissues (5 mice/group). Data are reported as means±SD. **P<0.01, ***P<0.001, and ****P<0.0001 unpaired *t*-test. ns: not significant. siMMP-12: siRNA silenced MMP-12; siNT: non-targeted siRNA.

To evaluate the function of MMP-12 in tumor growth *in vivo*, we established a CRPC xenograft nude mouse model, as shown in Figure 4A. Three weeks after the injection of C42B and PC3 cells into the subcutaneous region of the mice, which contains fat tissue, the tumor weight and volume of the mice in the siMMP-12 group were significantly reduced compared with those of the mice in the control and mock groups (Figure 4B and C, Supplementary Figure S1A and B). Consistent with the changes in the molecular pathways of C42B cells *in vitro*, mice in the siMMP-12 group showed greater levels of the autophagy protein LC3 than those in the control group, indicating that tumor growth was reduced (Figure 4D). Immunoblotting experiments were performed to measure protein expression in tumor tissues, and the results showed that the expression of the autophagy-related protein LC3B was increased and that of P62 was decreased significantly in the MMP-12-knockdown group. Additionally, the protein levels of CPT1 and CD36 were decreased in MMP-12-knockdown tumor cells (Figure 4E). Moreover, the expression of LC3B was increased in the siMMP-12-PC3 tumor tissues (Supplementary Figure S1C). These data suggest that MMP-12 affects CRPC tumor growth *in vivo* by regulating autophagy and lipid utilization.

**Figure 5:**
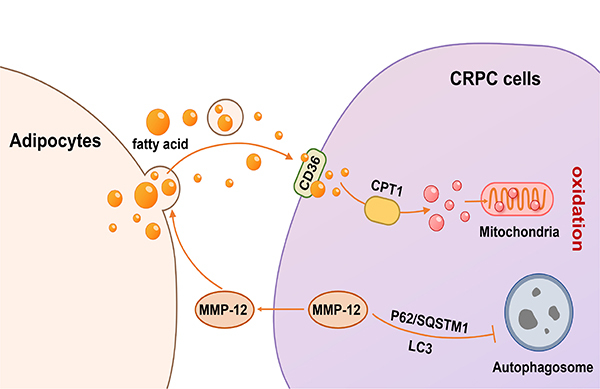
Potential mechanism of MMP-12 promoting the progression of castration-resistant prostate cancer (CRPC) cells. MMP-12 secreted by tumor cells might interact with surrounding adipocytes, leading to the release of free lipids, thereby promoting the uptake and utilization of lipids by tumor cells. MMP-12 might also alternatively restrain the autophagy process of cancer cells, thus promoting the migration and invasion of CRPC cells.

Overall, the downregulation of MMP-12 delayed CRPC cell migration and invasion as well as tumor growth by promoting autophagy and inhibiting lipolysis. In the peritumoral space, MMP-12 derived from CRPC cells may interact with adipocytes to facilitate adipocyte delipidation, thus promoting lipid uptake by CRPC cells (Figure 5).

## Discussion

The development and occurrence of malignant tumors, including lung cancer, epithelial ovarian cancer, and breast cancer, are closely associated with MMP-12 expression (22,23). MMP-12 is mainly secreted by inflammatory cells, such as monocytes and macrophages, but it is expressed at low levels or is not expressed at all in normal tissues (11). As a major regulator, MMP-12 is involved in tumor growth, migration, invasion, and immune escape, but only a few studies have evaluated the effects of MMP-12 on prostate cancer progression (12-14). MMP-12 was identified by gene and protein expression analysis to be a key protein that is associated with osteoblasts or osteolytic bone responses in patients with bone metastases from prostate cancer (24). An increase in the level of aromatase in PC3 cells can promote an increase in endogenous estrogen levels and enhance the expression of MMP-12 through the ERα pathway, thus promoting tumor metastasis (25). These results reflect the role of MMP-12 in CRPC to some degree. In this study, we found that the MMP-12 protein is highly expressed in human CRPC tissues and is positively correlated with an increase in the cancer Gleason score. Moreover, MMP-12 was highly expressed in the C42B and PC3 cell lines, further demonstrating its involvement in the progression of CRPC.

The periprostatic space, which is surrounded by adipocytes, may perform an endocrine function in malignant tumor behavior. Cancer cells can cause adipocytes to become smaller, disperse lipid droplets, and acquire a fibrous phenotype, forming tumor-associated adipocytes; additionally, these adipocytes secrete large amounts of cytokines and provide energy for tumor proliferation and metastasis and thus increase tumor malignant potential (26,27). Therefore, we focused on the relationship between MMP-12 in cancer cells and adipocytes. Since MMP-12 was not expressed in 3T3-L1 preadipocytes or mature adipocytes, we established a cell co-culture model *in vitro*. The number of mature adipocytes was increased in co-cultures with MMP-12-knockdown CRPC cells compared with MMP-12-expressing CRPC cells, suggesting that high expression of MMP-12 in cancer cells may interfere with the process of peripheral adipocyte maturation. MMP-12 plays an important role in macrophage-mediated extracellular matrix proteolysis and tissue invasion, and an increased number of macrophages in adipose tissue in obese mice participates in activating the inflammatory response (28,29). Moreover, via PCR and IHC analyses, several studies have shown that MMP-12 is produced by adipose tissue in obese mice (30,31). However, the western blotting results in our study showed that the expression of MMP-12 in mature adipocytes was low and was considered to be negative. The difference in these results may be related to differences in the research objects, specimen sources, and detection methods used.

MMP-12 has been found to be involved in the development and occurrence of malignant tumors (32). In patients with lung adenocarcinoma (LAC), the expression of MMP-12 is significantly increased in cancer tissues, and MMP-12 expression is closely related to the pathological stage and lymph node metastasis of LAC patients. Moreover, down-regulation of MMP-12 expression can inhibit the proliferation and invasion capacity of LAC cells (33). Our data also showed that the migration and invasion of cancer cells were significantly reduced after MMP-12 was silenced, which indicated that MMP-12 may promote the migration and invasion of CRPC cells. ECM cells, such as adipocytes, fibroblasts, immune cells, and vascular endothelial cells, form a complex tumor microenvironment that can affect tumor development. Among these cells, adipocytes promote the migration and invasion of tumor cells by secreting lipids, free fatty acids, and cytokines, leading to the malignant progression of tumors (7). PPAT contains androgens and their precursors provide stable and reliable local exogenous hormones for prostate cancer and support its growth and metastasis. Moreover, enhanced lipid uptake and synthesis are two hallmarks of prostate cancer, and these processes are regulated by androgen signaling, which is critical for prostate cancer development (34). This bidirectional association between the PPAT and tumor cells may facilitate the progression of cancer. We found that cancer cell lipid catabolism was significantly weakened in the test group compared with the control group when cells were transfected with siMMP-12. This effect may result from MMP-12 promoting cancer cell acquisition of energy from surrounding adipose tissues, thus increasing cancer cell migration and invasion. Furthermore, the tumor invasion-related proteins MMP-7 and MMP-9 were decreased in the context of MMP-12 deficiency. These results suggested that MMP-12 promoted tumor progression possibly through regulating the activity of other MMP family members.

Autophagy is an adaptive response of cells to metabolic stress. When cells are deficient in nutrients or under stimulated stress, the level of autophagy increases accordingly to cope with adverse factors (35). Autophagy exerts dual effects on tumors: it can inhibit tumor growth as well as protect tumors against adverse factors (36). Autophagy is closely related to the progression of CRPC, and relevant studies have preliminarily shown that tumor growth is reduced and survival time is prolonged in a CRPC mouse model with autophagy-related protein deficiency (37). Fatty acids that are released by adipose tissue are the primary energy source for prostate cancer cells during the development of CRPC. Shi et al. reported that androgens can mediate autophagy in prostate cancer cells and increase intracellular lipid deposition to regulate cell growth (38). Singh et al. (39) identified the role of autophagy in lipid metabolism by revealing that the inhibition of autophagy triggers increased storage of triglycerides in lipid droplets in mouse hepatocytes. Our findings confirmed that MMP-12 can regulate lipid catabolism in CRPC cancer cells and affect their migration and invasion. The results also confirmed that the expression of autophagy-related proteins in cancer cells was enhanced after MMP-12 silencing, which led to weakened cancer cell malignancy and inhibited tumor growth; these results suggested that MMP-12 may affect the progression of CRPC by regulating autophagy. Autophagy may contribute to oncogene-induced senescence, which can lead to permanent cell cycle arrest and affect cancer cell proliferation (40). When autophagy is excessively activated, cells die due to excessive self-degradation, which is also an important feature of autophagy-related tumor inhibition. Because the pathological molecular mechanism of CRPC progression is complex, additional investigations are needed to reveal the relationships and critical pathways involved.

### Conclusions

Our results confirmed that the downregulation of MMP-12 inhibited lipid catabolism in CRPC cells and promoted autophagy, thus enhancing migration and invasion and promoting prostate cancer growth. These data suggested that MMP-12 could be a potential tumor biomarker and therapeutic target and provide a new potential direction for treating CRPC.

## Supplementary Material

Click here to view [pdf].
