# Supplementary material for: Silencing of matrix metalloprotease-12 delays the progression of castration-resistant prostate cancer by regulating autophagy and lipolysis
Source: Braz J Med Biol Res. 2024 Mar 18;57:e13351. doi: 10.1590/1414-431X2024e13351 (PMC10946229; doi:10.1590/1414-431X2024e13351)
Supplement: Supplementary file 1 [file 1414-431X-bjmbr-57-e13351-suppl.pdf]

Supplementary Material

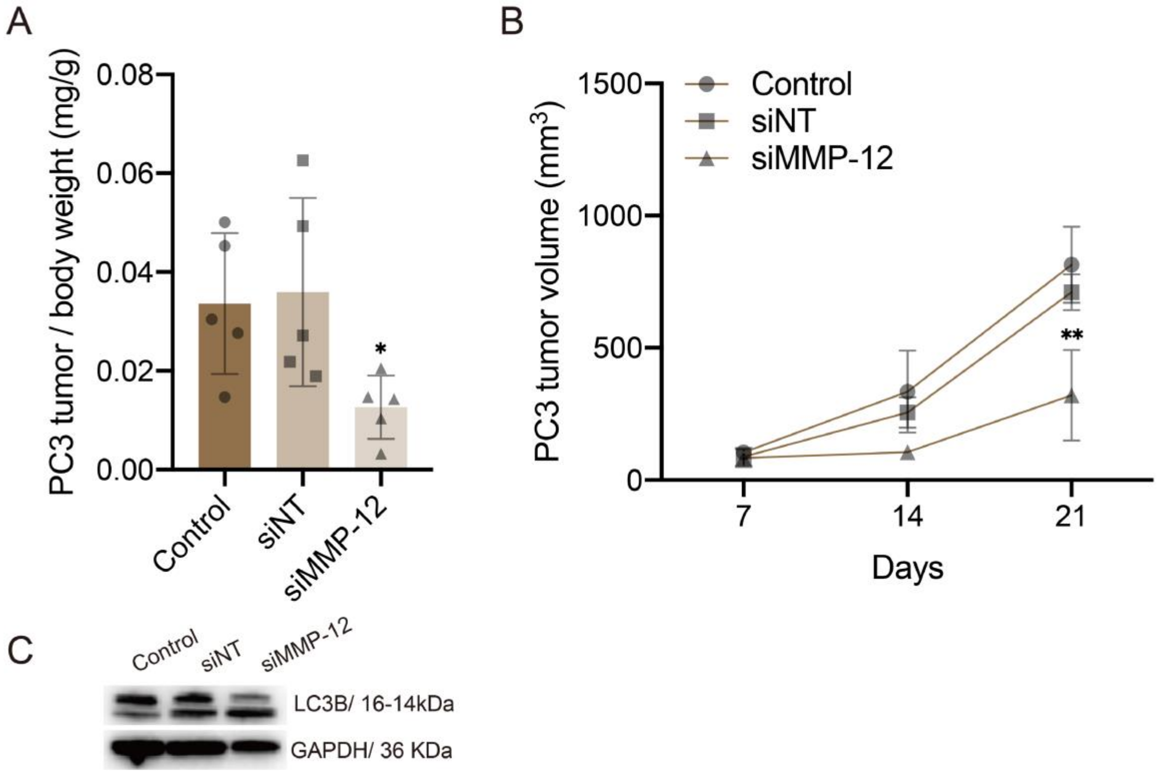

Figure S1. MMP-12 deficiency inhibited PC3 tumor growth. **A**, The relative tumor weight of different groups was measured at the end of the experiment. **B**, Tumor volume of different groups was measured on the 7th, 14th and 21st days. **C**, The autophagy-related protein LC3B was detected by western blotting in tumor tissues (5 mice/group). Data are reported as means±SD. *P<0.05 and **P<0.01 unpaired *t*-test. ns: not significant. siMMP-12: siRNA silenced MMP-12; siNT: non-targeted siRNA.
